# Preserving the marginal mandibular branch of the facial nerve during submandibular region surgery: a cadaveric safety study

**DOI:** 10.1186/s13037-018-0170-4

**Published:** 2018-08-23

**Authors:** Dimonge Joseph Anthony, Basnayaka Mudiyanselage Oshan Deshanjana Basnayake, Yasith Mathangasinghe, Ajith Peiris Malalasekera

**Affiliations:** Department of Anatomy, Faculty of Medicine, University of Colombo, Kynsey Road, Colombo 08, Sri Lanka

**Keywords:** Marginal mandibular branch, Facial nerve, Mandible, Submandibular incision

## Abstract

**Background:**

The marginal mandibular branch of the facial nerve is vulnerable to iatrogenic injuries during surgeries involving the submandibular region. This leads to significant post-operative morbidity. Studies assessing accurate anatomical landmarks of the marginal mandibular branch are sparse in South Asian countries. Present study was conducted to assess the relationship between the marginal mandibular branch and the inferior border of the body of mandible.

**Methods:**

Twenty-two preserved cadavers of Sri Lankan nationality were selected. Cadavers were positioned dorsal decubitus with necks in extension. The maximum perpendicular distance between the inferior/caudal most ramus of the marginal mandibular branch and the inferior border of the body of the mandible was recorded on both hemi faces.

**Results:**

Recorded maximum distance was 17.65 mm on left side and 10.80 mm on right side. Mean maximum distance, was 7.12 ± 2.97 mm. There was no statistically significant difference in the maximum deviation on left (7.84 ± 3.41 mm) and right sides (6.44 ± 2.37 mm).

**Conclusion:**

Course of the marginal mandibular nerve is complex. If the distance of the incision in the posterior submandibular approach is less than 2 cm from the inferior border of the mandible, there is a high probability of damaging the inferior ramus of the marginal mandibular branch of the facial nerve.

## Background

Facial nerve is the seventh cranial nerve, which supplies the muscles of the facial expression, sensory supply to the anterior two thirds of the tongue and secretomotor supply to the submandibular, sublingual and lachrymal glands. Marginal mandibular branch of facial nerve (MMBFN) is one of the main five terminal branches of the extracranial part of the facial nerve. It is given off within the substance of the parotid gland. There can be multiple rami of marginal mandibular nerve ranging from one to three [1].They run forwards and anteriorly towards the angle of the mandible deep to platysma and winds around the inferior border of the mandible. Then it turns upwards across the body of the mandible to pass under depressor angularis oris [2].It further divides to supply risorius and muscles of the lower lip and chin. Filaments given off at this point also communicate with the mental nerve [2].

Marginal mandibular nerve is at a vulnerable position during surgery of the area as it runs adjacent to the inferior border of the mandible. It is commonly damaged inadvertently at this site during surgeries involving submandibular region. Reported incidence of damage to the marginal mandibular nerve during submandibular gland removal is 0 to 20% [3–5]. But the true incidence is believed to be higher since most of these studies are retrospective and thus there is underreporting [6].

Iatrogenic trauma to the marginal mandibular nerve causes significant cosmetic disfigurement. Paralysis of the depressor anguli oris and the depressor labii inferioris cause inversion and flattening of the ipsilateral lip, thus disabling inferolateral movement [7].This results in an asymmetrical smile with elevation of the lower lip [7]. The deformity is more pronounced while the patient is crying [8]. This disfigurement leads to a significant perceived disability [6].

Hence, a sound knowledge regarding the surgical anatomy of marginal mandibular nerve at the upper neck in relation to the mandible is extremely important. Opinions in various textbooks diverge with regard to the relations of the marginal mandibular nerve [9]. Reported maximum downward deviation of this nerve from the inferior border of mandible is inconsistent (ranges from + 14 mm to + 40 mm in Caucasian populations [1, 10–15],-3.5 mm to + 30 mm in Mongoloid populations [16–20], + 0.8 to + 13 mm in American populations [21–24] and 2.3 mm in African populations [25]). The results of these studies are summarized in the Table 1.

**Table 1:** A summary of the studies describing the maximum downward deviation of the MMBFN from the lower border of the mandible (MMBFN – Marginal mandibular branch of the facial nerve)

Year of publication	Country	Sample size (hemifacies)	Maximum deviation of the MMBFN (mm)	Recommended distance for the incision from the angle of mandible (cm)	Special topographical findings
1996 [10]	Germany	55	14.00	2	MMBFN had up to 4 branches
2012 [11]	Turkey	44	40.08	None	Branches of MMBFN - one branch (36.4%), two branches 63.6%. Communications with the buccal branch was present in 4.6%
2007 [12]	Turkey	50	10.04	2	MMBFN ran above the inferior border of the mandible in 74%. In 22% of the times the MMBFN divided into two branches at the crossing point of the facial artery
2004 [13]	France	54	17.5	None	Single branch of MMBFN was found in 43%
1980 [15]	England	110	12.0	2	Communications between the MMBFN and the buccal branch were observed in 8%. Communications between the MMBFN and the cervical branch were observed in 12%.
2016 [16]	Korea	29	17.0	2	There were no significant differences of the observed distances in fresh and embalmed cadavers
2007 [18]	China	24	4.8	None	
1991 [19]	China	120	Between 2.1 and 3.0	3	Only 10% of the rami of the MMBFN ran below the lower border of the mandible
2009 [20]	Korea	85	19.8	Does not need to be far as 3–4 cm	

There is a scarcity of research evaluating the anatomical variations of the MMBFN from regional South Asian countries. Present study was conducted for the first time in a cohort of Sri Lankans to assess the relationship between MMBFN and the inferior border of the body of the mandible to make safe incisions when approaching submandibular region.

## Methods

The dissections were conducted on self-donated cadavers. The study was ethically approved by the Ethics Review Committee, Faculty of Medicine, Colombo and was conducted in accordance with the guidelines set forth by Declaration of Helsinky.

Twenty cadavers of male and female adults of Sri Lankan nationality were selected randomly. Cadavers with deformed submandibular region were excluded from the study. All selected cadavers were preserved with conventional arterial injection method using 10% formalin as the main preservative. Cadavers were positioned dorsal decubitus, with the neck in extension approximately 15 degrees so as to simulate surgical positioning.

**Fig. 1:**
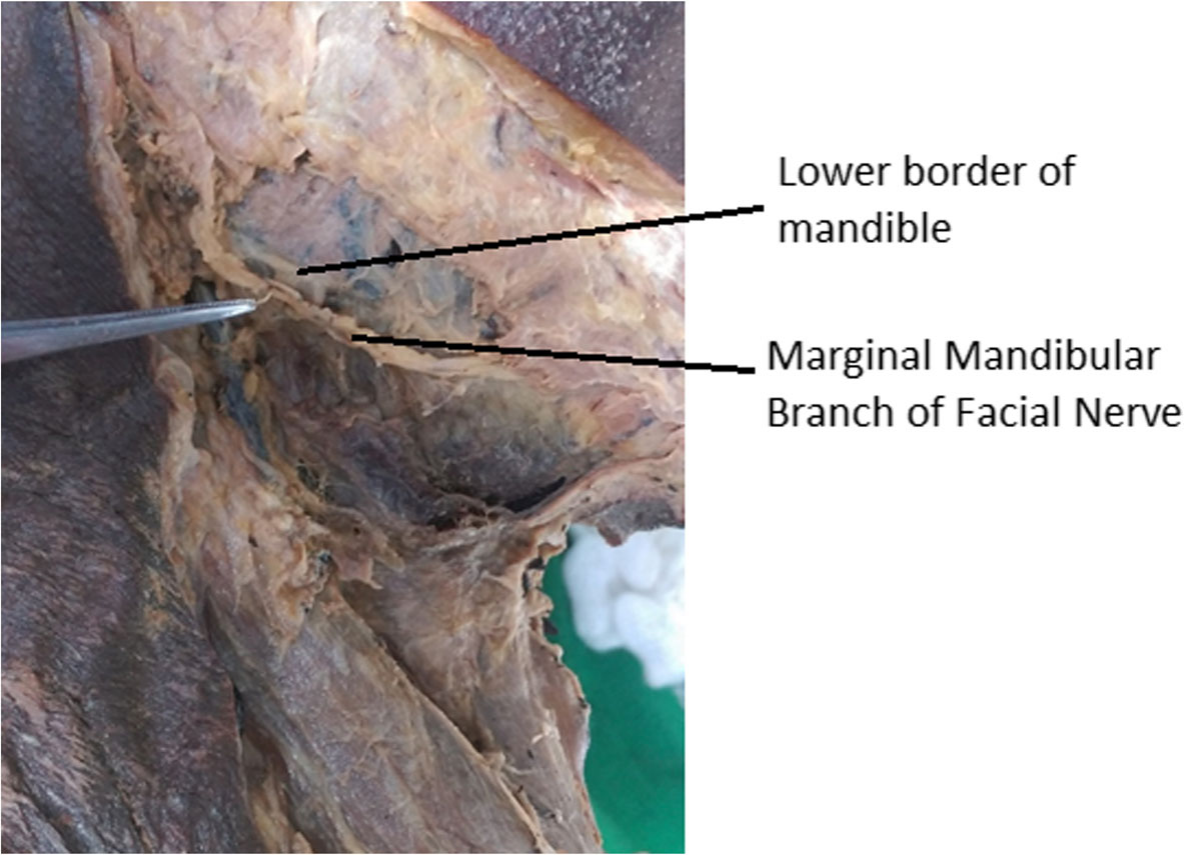
Relation between the marginal mandibular branch of facial nerve (MMBFN) and the lower border of the mandible

A skin incision was made 5 cm below and parallel to the lower border of mandible joining the anterior borders of sternocleidomastoid muscles on either side. A subplatysmal skin flap was made and reflected upwards to the lower lip. The marginal mandibular nerve was identified with careful blunt dissection under magnification and traced posteriorly to its origin within the substance of the parotid gland (Fig. 1). The nerve was left in place and dissected the superficial layers carefully to prevent mobilization of the nerve. Hence anatomical relations and distances to the adjacent structures were not disturbed. The perpendicular distance between the inferior border of the mandible and the inferior/caudal ramus of the marginal mandibular nerve was recorded on both sides. Measurements were recorded to the nearest 0.05 mm using a Vernier caliper [Manufacturer- Mitutoyo (Kanagawa- Japan) [Model No- 505-633-50]. If the inferior ramus was above the lower border of the mandible, the value was denoted with a minus, and vice versa. Data were analyzed using Statistical Package for Social Sciences (IBM SPSS) version 21 with a priori alpha of .05.

## Results

The MMBFN lied between the platysma and the investing layer of deep cervical fascia. The lowest ramus of MMBFN was located consistently below the lower border of the mandible in all the dissected hemifaces. Hence all the measured distances were positive. The distances were examined to determine the extent to which the assumption of normality was met. Skewness = 0.382 (SE = .365), kurtosis = 0.068 (.717) and the Shapiro-Wilk test = 0.974 (df = 42, *p* = .454) suggested that normality is a reasonable assumption.

Recorded maximum distance between the inferior border of the mandible and the caudal ramus of the marginal mandibular nerve on the left side was 17.65 mm. The next greatest deviation was 13.40 mm on the same side. The maximum distance on the right side was 10.80 mm. Mean maximum distance, was 7.12 ± 2.97 mm. An independent sample *t-*test showed that there was no significant difference in the maximum distance between the lowest ramus of the MMBFN and the lower border of the mandible on left (7.35 ± 2.63 mm) and right sides (6.44 ± 2.37 mm); *p* > .05. Ninety-five percent of the time, the outermost ramus of MMBFN lied within 6.09 mm to 7.65 mm distal to the inferior border of the mandible.

## Discussion

The results of the study demonstrated that all the rami of the MMBFN were situated within 17.7 mm and 10.8 mm from the inferior angle of mandible on the left and right sides respectively. Even though the results suggested an asymmetrical distribution of the rami, it was not statistically significant. This asymmetry was noted in a study conducted by Saylam et al. [12].Similarly, there was no statistically significant difference in two sides in that study [12]. In contrast to the majority of scientific literature,the lowest rami of MMBFN were located consistently below the lower border of the mandible [10, 12, 17, 18, 20–22, 26]. However this was in corroboration with a French study [27].

If the distance of the incision in the posterior submandibular approach is less than 2 cm from the inferior border of the mandible, there is a high probability of damaging the inferior ramus of the MMBFN.

This safe distance has been the subject of debate for many years. The classical teaching has been to make the incision two finger breaths distal to the angle of mandible. This arbitrary cutoff has been refined by a number of studies. The safe distances that can be derived from the available scientific publications are 10 mm [1], 20 mm [16, 23, 28], 30 mm [10, 19, 21] and 40 mm [11]. A three centimeter distance was described in Risdon’s technique [29, 30], which is a common approach in operative surgical procedures involving the submandibular region. But Baker and Conley found that the offshoots of MMBFN could be located 30 to 40 mm below the lower border of the mandible in lean subjects [14]. A summary of these studies are shown in Table 1. To date there is no guideline on this safe distance.

Special emphasis has been given to the positioning of the cadaver in similar research. We positioned the patient dorsal decubitus, with the neck in extension similar to position during surgery. This provides the surgeon with a good view. Hwang et al. found that the angle of neck extension does not change the position of the inferior ramus of the MMBFN with regard to the position of the surgical incision [17]. But surgeons should be aware that this relationship is liable to change if there is rotation of the neck [17].

Numerous studies have estimated the mean distance between the angle of mandible and the MMBFN. The objective of our study was to assess the maximum distance between the above mentioned structures for a safe incision. Therefore, describing the topographical variations of all the rami of MMBFN was out of scope of this study. Yang et al. described that there were no significant anatomical differences of MMBFN in fresh and embalmed cadavers [16]. Yet, we cannot rule out the possibility of such changes. Though we describe the maximum deviation of the nerve as a safe distance to make the incision, a more distally situated incision might be necessary to prevent occurrence of neuropraxia.

## Conclusion

The course of the marginal mandibular nerve is subjected to a wide array of anatomical variations in the submandibular region. Surgical anatomy of this nerve might vary among different populations. Appreciation of these variations is important to reduce morbidity following surgeries involving submandibular region. Different from earlier reports, we found at least one ramus of each MMBFN located caudal to the inferior border of the body of the mandible. MMBFN is at highest risk of being damaged when a skin incision is placed 6.09 to 7.65 mm from the inferior angle of mandible.

Since South Asian countries have heterogeneous populations, detailed anatomic dissections in this context is needed to understand the subtle variations of MMBFN in a surgical point of view. A systematic review of available publications is necessary before guidelines are set. Neurophysiological studies are needed to confirm whether the results of the cadaveric studies could be directly applied on clinical grounds.

## Abbreviation

MMBFN: Marginal Mandibular Branch of Facial Nerve
